# Long-Term Risperidone Treatment Induces Visceral Adiposity Associated with Hepatic Steatosis in Mice: A Magnetic Resonance Approach

**DOI:** 10.1155/2014/429291

**Published:** 2014-04-27

**Authors:** Florent Auger, Patrick Duriez, Françoise Martin-Nizard, Nicolas Durieux, Régis Bordet, Olivier Pétrault

**Affiliations:** ^1^Université Lille Nord de France, 59000 Lille, France; ^2^Département de Pharmacologie Médicale, CHULille, EA 1046, 59000 Lille, France; ^3^Inserm, U837, 59000 Lille, France; ^4^IMPRT-IFR114, 59000 Lille, France; ^5^Faculté de Pharmacie, 59006 Lille, France; ^6^Inserm, U1011, 59000 Lille, France; ^7^Institut Pasteur de Lille, 59019 Lille, France; ^8^UArtois, 62300 Lens, France; ^9^Laboratoire de Pharmacologie Médicale, EA 1046, Faculté de Médecine, Université Lille2, Pôle Recherche, 1 Place de Verdun, 59045 Lille Cedex, France

## Abstract

Although atypical antipsychotic drugs (APDs) have led to significant advances in the treatment of psychotic disorders, they still induce metabolic disturbances. We aimed at characterizing the metabolic consequences of a risperidone treatment and at establishing a link with noninvasive MR markers, in order to develop a tool for predicting symptoms of the metabolic syndrome. Fat deposition and liver morphometry were assessed by T1-weighted imaging. Fatty acid composition and fat accumulations in tissues were determined using MR spectroscopy with and without water suppression, respectively. Risperidone treatment induced a weight gain accompanied with metabolic disturbances such as hyperglycemic status, an increase in visceral adipose tissue (VAT), and liver fat depositions. Correlations using Methylene-Water Ratio (MWR) and Polyunsaturated Index (PUI) demonstrated a concomitant increase in the weight gain, VAT and liver fat depositions, and a decrease in the quantity of polyunsaturated fatty acids. These results were consistent with a hepatic steatosis state. We evaluated the ability of MR techniques to detect subtle metabolic disorders induced by APDs. Thus, our model and methodology offer the possibility to investigate APDs side effects in order to improve the health conditions of schizophrenic patients.

## 1. Introduction

Antipsychotic drugs (APDs) are widely used in current psychiatric practice and are commonly classified as typical (conventional) or atypical (second generation). Atypical APDs have been introduced in clinical practice after 1990, including clozapine, olanzapine, quetiapine, and risperidone. Atypical APDs cause less extrapyramidal symptoms (tremors) than typical APDs. However, both of them produce a weight gain [1–3], which increases the risk to develop a metabolic syndrome [4] associating several disorders such as diabetes mellitus, hypertension, hyperglycemia, dyslipidemia, and abdominal fat deposition [4, 5].

The excess of visceral adipose tissue (VAT) mass is particularly correlated to the prevalence of metabolic syndrome and insulin resistance [6]. Indeed, abnormal VAT depositions lead to the storage of lipids in undesired organs such as pancreas, skeletal muscle, heart, and liver. This so-called “ectopic fat deposition” contributes to the development of metabolic syndrome [6–12]. VAT accumulation could thus represent a biomarker of metabolic disturbances, as used in clinic through the measurement of waist circumference in replacement of the body mass index [5]. Nevertheless, the susceptibility to develop a metabolic syndrome is not systematically linked to risk factors as fat distribution in other compartments (i.e., visceral* versus* subcutaneous fat), insulin-resistance, or sedentary lifestyle [7–11]. The relative independence of these factors points out the difficulty to find reliable biomarkers. “Ectopic fat deposition” remains the most relevant process for diagnosing early metabolic disturbances, thus justifying the use of new noninvasive techniques to characterize body fat repartition.

Several techniques are widely used for the* in vivo* assessment of body fat, such as anthropometry [13], hydrodensitometry, air displacement plethysmography, bioelectric impedance, and dual energy X-ray absorptiometry [14, 15]. Their common limitation arises from their inefficiency in distinguishing subcutaneous adipose tissue (SAT) from VAT and in establishing an accurate body fat repartition. Computerized Tomography (CT) and Magnetic Resonance (MR) scans have the capability to distinguish SAT from VAT [16–21]. CT and MR techniques enable quantification of ectopic fat [16, 22–26]. MR imaging (MRI) and Magnetic Resonance Spectroscopy (MRS) are noninvasive techniques which are suitable for fat quantification and fat nature identification [27]. T1-weighted images and Dixon sequence are usually recorded to quantify SAT and VAT [7, 19, 26, 28]. They allow the segmentation of adipose tissues by dedicated posttreatment software [17, 29]. MRS enables the* in vivo *hepatic triglyceride (TG) quantification in order to diagnose hepatic steatosis in clinical practice. Due to its good correlation with histological data from liver biopsies [30], MRS is now considered as a reference for the quantification of noninvasive hepatic fat [7, 28].

Previous work showed the relevance of a mouse model for assessing weight gain induced by risperidone treatment [31]. In the present study, and for a translational purpose, we aimed at: (i) characterizing the metabolic disturbances associated with the weight gain induced by a long-term risperidone treatment and (ii) establishing a link with noninvasive MR markers, in order to develop a tool for predicting symptoms of the metabolic syndrome.

## 2. Materials and Methods

### 2.1. Animals

The Ethic community approved all protocols for animal experiments. Twelve seven-week- old female mice of C57BL/6N strain were purchased from Charles River laboratories (L'Arbresle, France).

### 2.2. Drug Treatment

Twenty-four female mice were examined in this study. Two groups of twelve animals were randomized by weight. One group was treated weekly over 24 weeks with intraperitoneal (IP) injection of 12.5 mg kg^−1^ of long-acting risperidone (Risperdal Consta, Janssen, USA) (risperidone group). The other group received IP injections of the vehicle of long-acting risperidone (control group). Mice were housed in a cage and maintained under a 12 h light-12 h dark cycle with free access to food and water. MR examinations were performed on the twenty-four mice. Pelleted food was weighted weekly to assess total grams of food consumed per cage and per day, and then divided by six to estimate the average weekly food consumption per mouse. Following MR examinations, the twenty-four mice were sacrificed by an overdose of pentobarbital. Biological samples were collected for histological examination.

### 2.3. MRI

All MR examinations were done after the 24 weeks of risperidone treatment on a 7 T Bruker Biospec (Ettlingen, Germany) imaging system equipped with a 40 cm horizontal bore magnet. Anesthesia was induced by 2% isoflurane and maintained at 1%–1.5% along acquisition, depending on respiration frequency. A pneumatic pillow (SA Inc. Stony Brook, NY) was used to perform and monitor triggered respiratory gating. Each mouse was placed in a cylindrical coil (39 mm inner diameter). Multislice gradient echo sequence was triggered with respiration and performed to assess mouse position inside the magnet, with the following sequence parameters: TR/TE (Repetition Time/Echo Time) = 200/3 ms, FA (Flip Angle) = 30°.

**Figure 1:**
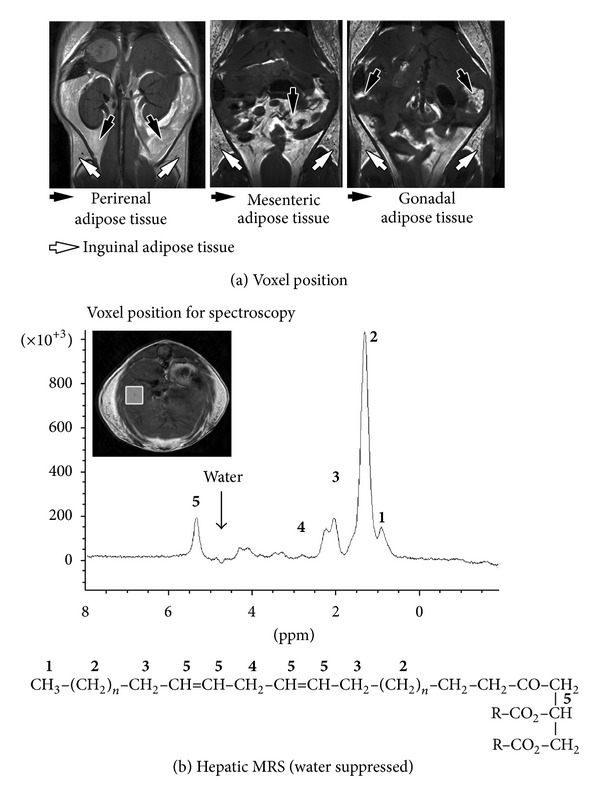
Hepatic lipids composition. (a) Three coronal T1-weighted MR images displaying inguinal, perirenal, mesenteric, and gonadal adipose tissues. These images were used to perform volumetric analyses. (b) Axial T1-weighted liver image with the voxel position used for MRS analyses and an example of a water suppressed MRS spectrum. Each peak corresponds to a particular chemical group described as follows:** 1** = terminal methyl (CH_3_) at 0.9 ppm;** 2** = methylene groups of saturated fatty acids and the “saturated” components of mono- and poly fatty acids (CH_2_)_*n*_ at 1.3 ppm;** 3** = allylic as the methylene adjacent to methene groups (CH_2_) at 2.1 ppm;** 4** = diallylic as the methylene inserted between sequential methene groups of fatty acid (CH–CH_2_–CH) at 2.8 ppm;** 5** = methene as the olefinic groups (CH=CH) at 5.3 ppm.

Liver and adipose tissue images were acquired by T1-weighted imaging, using axial and coronal Rapid Acquisition with Relaxation Enhancement (RARE) sequence, which was gated with respiration. Multislice coronal (FOV = 3.6 × 3.6 cm, 14 contiguous slices of 1.5 mm thick) and axial (FOV = 3 × 3 cm, 18 contiguous slices of 2 mm thick) RARE images were collected with a TR of 400 ms, a TE of 9 ms, a 256 × 256 data matrix, and a number of repetitions (NEX) of 2. Reconstructions were done to evaluate the volumes of liver, Inguinal Adipose Tissue (IAT, characterizing the subcutaneous compartment), and VAT (defined as the sum of perirenal fat, gonadal fat, and mesenteric fat, Figure 1(a)). Reconstructions were manually drawn on ITKSnap software [32] for each set of T1-weighted images.

### 2.4. MRS

Single-voxel localized 1H MR spectra were acquired using the PRESS sequence (respiratory gated) with the following parameters: voxel volume 3 × 3 × 3 mm; TR = 10,000 ms; TE = 20 ms. To avoid obvious blood vessel and subcutaneous fat contributions, the voxel was carefully placed on previous anatomic axial and coronal T1-weighted images (Figure 1(b)). The magnetic field homogenization remains a critical step, and the local shimming was performed until the water line width achieves less than 50 Hz conditioning the spectral resolution. Spectral data were processed on Topspin 2.0 (Bruker, Germany).

### 2.5. Methyl Water Ratio (MWR)

The MWR was assessed from localized proton spectroscopy performed without water suppression.* In vivo, *hepatic fatty acid proportion is determined as the percentage of the bulk methylene resonance to water [33]. So, MWR is defined as the ratio of the intensity of the lipid proton peak (methylene bulk) with the intensity of the water proton peak, allowing the estimation of the quantity of intracellular fat accumulation in the tissue.

### 2.6. Lipid MR Spectroscopy

The localized proton spectroscopy was acquired with manual water suppression using VAPOR (VAriable Power and Optimized Relaxation delays) sequence module and a signal accumulation amounted to 64. The different ratios were calculated from peak intensities of the lipid spectra. Previous works reported that the T1 relaxation values of liver methylene and methene groups were respectively between 0.39 to 1.20 s and 0.60 to 1.16 s [34, 35]. In order to fully relax all resonances (up to 5 × T1), we set the TR parameter of the PRESS sequence to 10 s and no further T1 correction was necessary. Depending on spectral resolution, the MR lipid spectrum profile is characterized by nine peaks but only five of them were usually considered for the lipid index calculation (see Figure 1(b)):** 1** = terminal* methyl* (CH_3_) at 0.9 ppm;** 2** =* methylene* groups of saturated fatty acids and the “saturated” components of mono- and poly fatty acids (CH_2_)_*n*_ at 1.3 ppm;** 3** =* allylic* as the methylene adjacent to methene groups (CH_2_) at 2.1 ppm;** 4** =* diallylic* as the methylene inserted between sequential methene groups of fatty acid (CH–CH_2_–CH) at 2.8 ppm;** 5** =* methene* as the olefinic groups (CH=CH) at 5.3 ppm.

Calculation of hepatic lipid indexes was based on the proportion of constitutive saturated mono- and poly unsaturated fatty acids according to Johnson and colleagues' study [36]. Using MR spectroscopy, four indexes can be calculated: the unsaturated index (UI), the unsaturated index surrogate (UIs), the saturated index (SI), and the polyunsaturated index (PUI). The formulas were as follows:

(1) $$\begin{array}{c} \text{UI}=\frac{I_{\text{methene}}}{I_{\text{methene}}+I_{\text{allylic}}+I_{\text{methylene}}+I_{\text{methyl}}}, \end{array}$$

where *I*_methene_, *I*_allylic_, *I*_methylene_, and *I*_methyl_ are, respectively, the signal amplitude of the methene, allylic methylene, bulk methylene, and terminal methyl peaks.

UIs formula is based on the principle that methene functional group is always next to allylic methylene group in monounsaturated fatty acids and with both allylic and diallylic methylene groups in Poly Unsaturated Fatty Acids (PUFAs). Thus UIs formula is as follows:

(2) $$\begin{array}{c} \text{UIs}=\frac{I_{\text{allylic}}+I_{\text{diallylic}}}{I_{\text{allylic}}+I_{\text{diallylic}}+I_{\text{methylene}}+I_{\text{methyl}}}, \end{array}$$

where *I*_diallylic_ is the signal amplitude of the diallylic methylene peak. SI is calculated as the complemented index of UI_s_ and can be easily assessed as follows:

(3) $$\begin{array}{c} \text{SI}=1-\frac{I_{\text{allylic}}+I_{\text{diallylic}}}{I_{\text{allylic}}+I_{\text{diallylic}}+I_{\text{methylene}}+I_{\text{methyl}}}. \end{array}$$

Lastly, the diallylic peak is detected only in PUFAs (18 : 2, 18 : 3, 20 : 4, 20 : 5, and 22 : 6) in liver. Thus the measure of PUI is

(4) $$\begin{array}{c} \text{PUI}=\frac{I_{\text{diallylic}}}{I_{\text{allylic}}+I_{\text{diallylic}}+I_{\text{methylene}}+I_{\text{methyl}}}. \end{array}$$

As the methene signal could not be correctly detected when water suppression pulses are applied, we decided to omit UI according to the method described by Johnson and colleagues [36] and the unsaturated index was therefore calculated with UIs. A spectrum with a low signal noise ratio (<2) for the diallylic peak at 2.8 ppm was excluded from the study.

### 2.7. Spontaneous Locomotor Activity

Spontaneous motor activity was measured using an actimeter (Panlab, Barcelona, Spain). This apparatus allowed horizontal motor activity (traveled distance, in centimeters), rearing behavior (number of rears), and mean velocity (in centimeters per second). These parameters gave clues about ability of mice to explore a new environment. Motor activity was recorded for ten minutes.

### 2.8. Glucose Tolerance Test

After fasting in fresh cages for 6 hours, approximately 25 *μ*L of tail tip blood was collected to assess fasting glucose levels. Thereafter, mice received 2 g/kg of 15% dextrose by ip injection. Sample tail tip blood glucose levels were measured using a glucometer (Accu-Check performa, Roche Diagnostics GmbH, Mannheim, Germany) at 10, 20, 30, 60, 90, and 120 min.

### 2.9. Histology

IAT and livers were collected and weighted. Livers were then fixed in 4% paraformaldehyde for Oil Red O (ORO) staining. Analysis of lipid deposition was made by ORO staining on 7 *μ*m-thick frozen-liver sections, using Harris haematoxylin for nucleus coloration. The surface of liver lipid droplets was quantified using image J software (1.43, Wayne Rasband, NIH, USA). Lipid droplet volumes were calculated from the surface previously measured by the software. Results were presented as a volume variation relative to the control group.

### 2.10. Assays

#### 2.10.1. Plasma

Dosage of cholesterol and TG was performed immediately after MR. Plasma levels of total cholesterol (TC), TGs, and high density lipoprotein cholesterol (HDLc) were determined using commercially available kits (BioMérieux, France). Non-HDL cholesterol (Non-HDLc) was calculated by subtraction of HDLc from TC.

#### 2.10.2. Livers TG

Frozen liver tissue (50 mg) was homogenized in SET buffer (1 mL; sucrose 250 mM, EDTA 2 mM, and Tris 10 mM), followed by two freeze-thaw cycles and three times passages through a 27-gauge syringe needle, and a final freeze-thaw cycle. Protein content was determined using the BCA method (Interchim, France), and TG and cholesterol were measured as described above.

### 2.11. Statistical Analyses

All statistical analyses were performed using StatWiev software (SAS institute Inc., Version 5.0). Data are reported as mean ± standard deviation (SD).The criterion for statistical significance was *P* < 0.05.

Statistical analyses were performed as follows.

1. When distributions were normal in each group, a Student's *t*-test was used. If one or more group was not normally distributed, the nonparametric Mann-Whitney was used.
2. To assess differences on autopsy, MR volumetry, and MRS experimental datasets, we performed ANCOVA tests using mice weight as a covariate in order to adjust statistical analyses and suppress the influence of the weight gain.
3. For correlations among data, a Spearman test was used.
4. A two-way ANOVA was performed to test weight gain and food intake.

## 3. Results

### 3.1. Risperidone Induced a Long-Term Weight Gain in Mice

**Figure 2:**
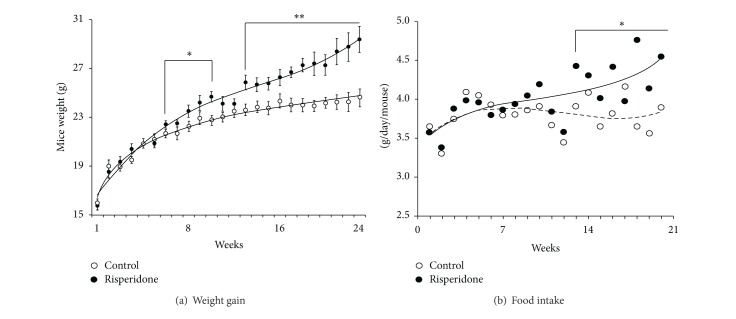
Twenty-four week risperidone treatment increased mice weight and food intake. (a) Effect of risperidone on weight gain in female mice. Differences between both groups appeared significant after 7 weeks of treatment and remained significant at the end of the experiment (24 weeks: control (∘) 24.58 ± 1.21 g* versus* risperidone (•) 31.33 ± 2.42 g; *P* < 0.01, *n* = 24). (b) Effect of risperidone on food intake. Risperidone treatment increased food intake after 13 weeks (Control: 3.90 ± 0.17 g/day/mouse* versus* Risperidone: 4.43 ± 0.47 g/day/mouse *P* < 0.05; *n* = 24).

After 24 weeks, a greater weight gain was observed in mice treated by risperidone compared to the control mice. At the beginning of the experiment, the mean weight of the risperidone group was 16.75 ± 0.87 g and 16.00 ± 1.04 g for the control group to achieve, respectively, 31.33 ± 2.42 g* versus *24.58 ± 1.21 g (*P* < 0.01, *n* = 24) at the end of the experiment (Figure 2(a)). In addition, this weight gain was associated with a change in food intake in treated animals from the 13th week (Control: 3.90 ± 0.17 g/day/mouse* versus* Risperidone: 4.43 ± 0.47 g/day/mouse; *P* < 0.05; *n* = 24Figure 2(b)) as well as with a modification in spontaneous locomotor activity (Table 1). Indeed, the treatment induced a reduction of the distance covered in the arena (Control: 6, 300 ± 687 cm* versus* Risperidone: 5, 041 ± 688 cm *P* < 0.001; *n* = 24; Table 1), in association with a diminution of the mean velocity of animals (Control: 12.11 ± 1.55 cm·s^−1^ *versus* Risperidone: 9.54 ± 1.27 cm·s^−1^; *P* < 0.01, *n* = 24; Table 1). Moreover, the exploration behaviour was also modified by the treatment as revealed by the reduction of the rearing number (Control: 29.17 ± 10.07* versus* Risperidone: 65.83 ± 11.68; *P* < 0.01, *n* = 24; Table 1). These results suggest that the risperidone-induced weight gain could at least in part be linked to an increase of appetite combined with a reduced spontaneous locomotor activity.

### 3.2. Could the Risperidone-Induced Weight Gain Be Associated with Metabolic Disturbances?

**Figure 3:**
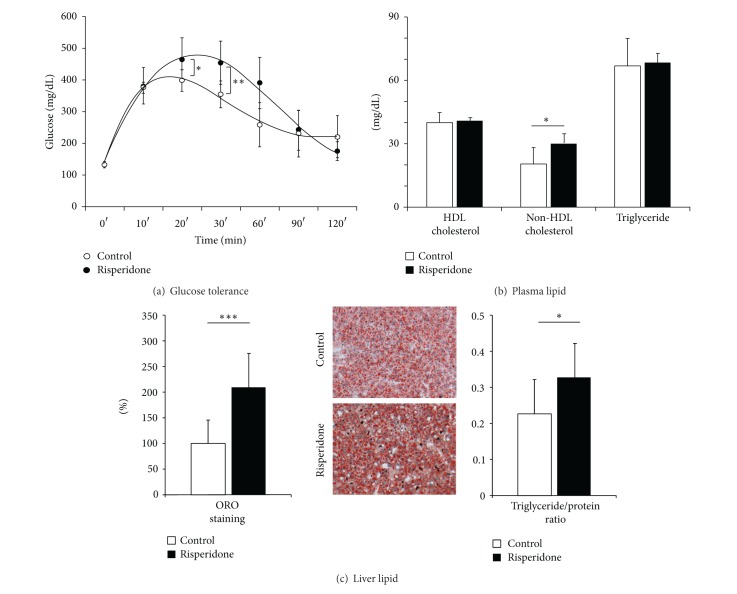
Metabolic disturbances induced by a 24-week treatment of risperidone on female mice. (a) Effect of risperidone on glucose tolerance in female mice. Risperidone treatment significantly decreased glucose tolerance. (b) Effect of risperidone on plasma lipid concentration. Risperidone treatment increased non-HDLc (26.90 ± 6.73 mg/dL* versus *18.82 ± 7.61 mg/dL; *P* < 0.01, *n* = 24). (c) Effect of risperidone on liver fat accumulation. Risperidone treatment induced an increase in liver lipid accumulation as displayed by ORO staining (control (□) 100.00 ± 46.58%* versus* risperidone (■) 210.96 ± 68.19%; *P* < 0.05, *n* = 21; left histogram and central pictures) and TG/protein ratio (control (□) 0.225 ± 0.097* versus* risperidone (■) 0.327 ± 0.096; *P* < 0.05, *n* = 24; right histogram).

We sought several metabolic disorders such as glycemic status, blood lipid analysis, and specific biological samples as the liver and fat depositions in order to identify peripheral consequences of the weight gain induced risperidone. The glucose tolerance test revealed that risperidone led to a hyperglycemic status as shown by the glucose tolerance test (Figure 3(a)) and by the area under the curve values (AUC: 27453 ± 2389  (mg/dL·120 min)* versus *42276 ± 12999  (mg/dL·120 min); *P* < 0.01, *n* = 20; Table 2). Blood analysis showed that risperidone increased the non-HDLc level compared to control mice (26.90 ± 6.73 mg/dL* versus *18.82 ± 7.61 mg/dL; *P* < 0.01, *n* = 24). But no significant difference was found concerning fasting TG (78.07 ± 30.07 mg/dL* versus *66.21 ± 11.18 mg/dL, *P* = 0.05637, *n* = 24) and HDLc concentrations (44.20 ± 6.77 mg/dL* versus *42.59 ± 5.121 mg/dL, *P* = 0.8625, *n* = 24; Figure 3(b)).

To test whether the risperidone treatment modified the fat deposition, we quantified subcutaneous inguinal and visceral fat from anatomic MR images as defined previously (Figure 1). Table 2 summarizes the volumetric analyses. The IAT and VAT volumes were, respectively, 2-fold and 3-fold higher in risperidone-treated mice than in the control group (IAT: 544.19 ± 127.20 mm^3^ *versus *232.40 ± 34.20 mm^3^ and VAT: 1065.06 ± 383.76 mm^3^ *versus *317.89 ± 116.79 mm^3^; *P* < 0.001, *n* = 24; Table 2). In addition, the best correlation between the adipose tissue accumulation and the glucose tolerance test was interestingly found for the VAT (correlation factor = 0.867, *P* < 0.001, *n* = 20; Table 3), suggesting that VAT could represent a good biomarker of the hyperglycemic status in our experimental model.

At the liver level, the autopsies revealed that risperidone treatment increased the liver weight (1.50 ± 0.17 g* versus *1.10 ± 0.14 g; *P* < 0.001, *n* = 24; Table 2). This hepatomegaly was confirmed by the volumetric data from MRI, as demonstrated by the significant increase in liver volume after risperidone treatment (702.77 ± 32.01 mm^3^ *versus *635.74 ± 49.54 mm^3^; *P* < 0.01, *n* = 24; Table 2). The ORO staining quantification showed a significantly higher lipid accumulation in the liver of mice treated by risperidone than in the control mice (100.00 ± 46.58%* versus *210.96 ± 68.19%; *P* < 0.001, *n* = 21; Figure 3(c), left panel). Results from hepatic lipid analyses were expressed as the ratio of TG to the protein concentration. The risperidone treatment increased the TG/protein ratio, pointing out an intrahepatic accumulation of TG (0.327 ± 0.096* versus *0.225 ± 0.097; *P* < 0.05, *n* = 24; Figure 3(c), right panel). This histological pattern clearly shows an early hepatic steatosis.

### 3.3. Quantification of the Hepatic Fat by MR Spectroscopy

**Figure 4:**
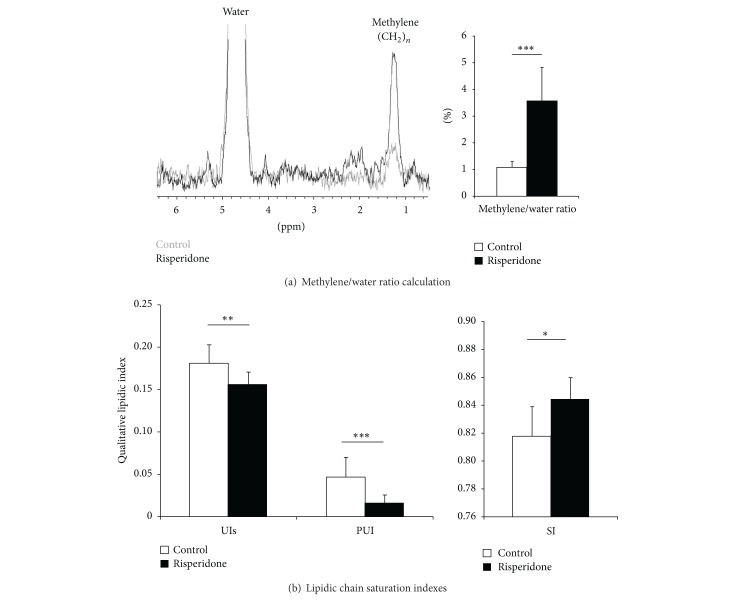
Quantitative and qualitative* in vivo* lipid assessments. (a) Representative of water suppressed MR liver spectra (left; control: grey spectrum; risperidone: black spectrum). Spectra resulted from a voxel analysis positioned in liver as described in Figure 1(b). Water and methylene peaks were clearly visible and enabled the assessment of the methylene water ratio (MWR). MWR revealed that risperidone treatment increased the lipid accumulation in liver (right histogram: control (□) 1.10 ± 0.20%* versus* risperidone (■) 3.57 ± 1.26%; *P* < 0.001, *n* = 24). (b) Unsaturated Index surrogate (UIs), Poly Unsaturated Index (PUI), and Saturated Index (SI) reflecting the hepatic lipid composition. Compared to control mice, risperidone significantly induced an increase in UIs (control (□) 0.156 ± 0.016* versus* risperidone (■) 0.182 ± 0.021; *P* < 0.01, *n* = 20), in SI (control (□) 0.844 ± 0.016* versus* risperidone (■) 0.818 ± 0.021; *P* < 0.05, *n* = 20), and a decrease in PUI (control (□) 0.047 ± 0.023* versus* risperidone (■) 0.017 ± 0.008; *P* < 0.001, *n* = 20).

Spectroscopy can easily distinguish the quantities of water and fat in body tissues. This technique is based on the calculation of the methylene/water ratio (MWR; Figure 4(a)). The MWR values calculated from hepatic spectra were significantly higher in the risperidone-treated group than in the control group (3.57 ± 1.26%* versus *1.10 ± 0.20%; *P* < 0.001, *n* = 24; Figure 4(a), histogram). This result was in good accordance with the histological pattern, pointing out the high sensitivity of the MRS technique for the detection of fat accumulation in the liver. We drew a correlation between the MWR/TG protein ratio (correlation factor = 0.798, *P* < 0.001, *n* = 24; Table 3) and ORO staining proportion (correlation factor = 0.747, *P* < 0.01, *n* = 20; Table 3), also found proportional to the fat accumulation in the liver.

Table 3 summarizes the link between MRS, MRI, and the postmortem data. We found a strong correlation between MWR/VAT volume (correlation factor = 0.798, *P* < 0.001, *n* = 24; Table 3) and MWR/mice weight (correlation factor = 0.859, *P* < 0.001, *n* = 24; Table 3). These correlations demonstrated a concomitant increase of weight gain, fat accumulation in the liver, and VAT deposition.

### 3.4. Qualitative Aspects of the Lipid MR Spectrum

Given the limit of 50 Hz for spectral resolution, 4 acquisitions had not been performed, 2 in each group. Therefore, 20 animals were considered in lipid indexes calculations described previously in the methods section and in Figure 1(b). In addition to the liver fat accumulation, the spectral analysis provides information on the realignment of the lipid chains. Compared to the control group, the risperidone treatment had an effect on UIs (Risperidone: 0.156 ± 0.016* versus* Control: 0.182 ± 0.021; *P* < 0.01, *n* = 20), on SI (Risperidone: 0.844 ± 0.016* versus* Control: 0.818 ± 0.021; *P* < 0.05, *n* = 20), and on PUI (Risperidone: 0.017 ± 0.008* versus* Control: 0.047 ± 0.023; *P* < 0.001, *n* = 20) as illustrated in Figure 4(b). The decrease in PUI and the concomitant increase in SI suggest a pathological state of the fatty acid availability in a suffering liver. The aggravation of hepatic steatosis was proportional to the decrease in PUI values. In our model, we found a closed correlation between PUI and mice weight (correlation factor = −0.868, *P* < 0.001, *n* = 20; Table 3).

## 4. Discussion

Two aspects are addressed in this study: first, our experimental model, which gradually developed a metabolic disturbance pattern associated with a long-term risperidone treatment, and second, our methodology, based on magnetic resonance, which evidenced that these noninvasive techniques were suitable for the detection of body fat alterations and the resulting hepatic steatosis.

In our experimental mouse model, the risperidone treatment induced a weight gain after 24 weeks. We concomitantly described here components of the metabolic syndrome [5] in mice treated by risperidone. Treated mice developed (i) a hyperglycemic status, (ii) an increase in non-HDL cholesterol plasma level, (iii) an abnormal deposition of fat in the inguinal and visceral areas, and (iv) a hepatomegaly related with early hepatic steatosis. The other advantages of this model are based on the risperidone-induced change in behaviour of the animal in terms of food intake and spontaneous locomotor activity. Several pieces of evidence support the idea that ADPs-induced weight gain would result from an increase in appetite self-mediated by a histaminic pathway at the CNS level [37]. Furthermore, risperidone could reduce locomotor activity by blocking the dopaminergic system [38, 39]. All these aspects highlight the relevance of our experimental model for the study of metabolic dark-side effects of ADPs.

The second aspect of this study is the use of noninvasive MR techniques. The volumetric analysis was performed through the use of T1-weighted imaging and was allowed the quantification of fat deposition in subcutaneous and visceral areas. Our findings showed a closed correlation between VAT and hyperglycemic status, in good accordance with clinical evidences suggesting a link between the prevalence of metabolic syndrome and an abnormal visceral adiposity [6]. This is why the different components of fat had to be distinguished. Previous clinical works reported that the abnormal VAT development led to lipid storage in undesired sites such as pancreas, skeletal muscle, heart, and liver [6, 7, 12]. In this study, we assessed the liver fat quantity reflected by MWR using MR spectroscopy and established a link between the mice being overweight, the increase in VAT, and liver fat deposition. These findings demonstrated that the MR methods constituted an accurate tool to study the possible fat reallocation from peripheral adipose tissue to the liver as suggested by clinical observations [40]. An interesting feature in our use of spectroscopy was the relatively low fat quantity in our model (MWR = 3.57 ± 1.26%) compared to other experimental models such as obese mice (ob/ob) or methionine choline deficient diet mice displaying a high fat level, respectively, MWR = 38% and MWR = 31% [41].

The low concentration of liver fat makes the hepatic steatosis difficult to diagnose by MR techniques. Clinical and preclinical studies showed modifications in nature and proportion of fatty acids in a suffering liver [42, 43]. The pathogenesis of steatohepatitis involves, at least in part, an oxidative process explaining the decrease in the PUFA level in steatotic liver. Indeed, assumptions indicate that polyunsaturated moieties within phospholipid membranes, the (*n*-3) species in particular, are most susceptible to free radical attacks leading to a reallocation of unsaturations [44, 45]. Using chromatographic techniques, two major markers have been identified as relevant for characterizing steatosis: the increase in saturated fatty acids and the depletion of polyunsaturated fatty acids [42, 46]. In our study, to validate the potential presence of this pathology, a water suppressed spectroscopy sequence was performed to estimate saturated and unsaturated fatty acid proportions. Compared to the control group, our spectrum analysis highlighted a significant decrease in PUI and an increase in SI in risperidone-treated mice. This result is in good agreement with previous works in which a similar variation of PUI was reported on obese patients with hepatic steatosis. This study concluded that hepatic steatosis potentiated the decrease in PUI [36]. Despite our less severe experimental model than an obesity model, our results clearly showed that the high sensitivity of the MR techniques enabled the diagnosis of early hepatic steatosis, as shown by the different correlations with PUI and MWR.

Nevertheless, these techniques have their own limitations, especially when the target organ for instance liver is subjected to respiratory motion. Although respiratory gating improves the quality of acquisitions, residual breathing artifacts may yield to uncertainty in amplitude and phase variations in localized MR spectra. These artifacts may induce bias on the spectral resolution which is dependent on the homogenization of the magnetic field (shim), and consequently modify the calculation of saturation/unsaturation indices. To minimize breathing artifacts, the ideal condition should define a TR value as close as possible to the steady state duration of breathing. Nevertheless, the use of low TR values leads to a T1-weighting at the expense of the signal/noise ratio. Compromisingly, we selected a high TR value to promote the signal/noise ratio. The magnetic field strength represents also a parameter which conditions a good signal/noise ratio, but depends only on the properties of the machine. The arrival of 3 T magnets for patients in clinical practice and 7 T magnets for clinical research may promote the use of these noninvasive techniques for diagnosis assistance through optimized signal/noise ratio and acquisition time.

Although our experimental model is far from obesity models, the subtle metabolic disturbances observed here demonstrated that MR approaches leave the opportunity to evaluate consequences of the risperidone treatment. We highlight three noninvasive markers to predict metabolic disturbances and hepatic steatosis severity when critical limits are reached and exceeded: mice weight up to 26 g, MWR up to 2.5%, and PUI down to 0.17. As a perspective to improve health conditions of schizophrenic patients, this experimental approach (model and methodology) could represent a relevant tool to (i) define the best effective dose of ADPs to maintain CNS effects and minimize metabolic dark-side effects; (ii) evaluate the benefit of a functional food such as dietaries supplemented with omega-3 [47] or curcuminoids [48]; (iii) develop new drugs through the validation of new therapeutic strategies using an active principle or a new pharmacological adjuvant targeting the CNS or not, in a view to reduce metabolic disturbances.

## 5. Conclusion

In this study, we proposed a pathophysiologic model of slight metabolic disturbances induced by a long-term risperidone treatment, which have been evaluated* in vivo* by MR methods. Three parameters have been selected: MWR, PUI, and mice weight as reliable factors to define the severity of the disturbances. Thus, our model and methodology offer the possibility to investigate APDs side effects more precisely, in order to improve health conditions of schizophrenic patients.

## Figures and Tables

**Table 1:** Spontaneous locomotor activity.

Data sets	Control group		Risperidone group		Significance
	*n*	Mean ± SD	*n*	Mean ± SD	
Distance (cm)	12	6300 ± 687	12	5041 ± 688	S (*P* < 0.001)
Mean velocity (cm·s^−1^)	12	12.11 ± 1.55	12	9.54 ± 1.27	S (*P* < 0.001)
Numbers of rearing	12	65.83 ± 11.68	12	29.17 ± 10.07	S (*P* < 0.001)

**Table 2:** Experimental parameters analysis.

Data Sets		Control group		Risperidone group		Significance
		*n*	Mean ± SD	*n*	Mean ± SD	
AUC (mg/dL·120 min)		10	27,453 ± 2,389	10	42,276 ± 12,999	S (*P* < 0.001)

Tissue weight (g)	Liver	12	1.10 ± 0.14	12	1.50 ± 0.17	S (*P* < 0.001)

Tissue volume (mm^3^)	Liver	12	635.74 ± 49.54	12	702.77 ± 32.01	S (*P* < 0.01)
	IAT	12	232.40 ± 34.20	12	544.19 ± 127.20	S (*P* < 0.001)
	VAT	12	317.89 ± 116.78	12	1,065.06 ± 383.76	S (*P* < 0.001)

**Table 3:** Correlations.

Correlations		*n*	*R* ^2^	Correlation factor	Significance
AUC/tissue volume	Liver	20	0.080	0.406	NS
	VAT	20	0.349	0.867	S (*P* < 0.001)
	IAT	20	0.269	0.772	S (*P* < 0.001)

MWR/hepatic lipid assay	TG/protein	24	0.440	0.798	S (*P* < 0.001)
	ORO staining	20	0.695	0.747	S (*P* < 0.01)

MWR/VAT		24	0.610	0.798	S (*P* < 0.001)

MWR/mice weight		24	0.651	0.859	S (*P* < 0.001)

MWR/AUC		20	0.611	0.798	S (*P* < 0.001)

PUI/mice weight		20	0.399	−0.868	S (*P* < 0.001)
